# Failure to progress

**DOI:** 10.7554/eLife.66254

**Published:** 2021-02-16

**Authors:** Duncan Greig, Jasmine Ono

**Affiliations:** Centre for Life’s Origins and Evolution, Department of Genetics, Evolution and Environment, University College LondonLondonUnited Kingdom

**Keywords:** nontransitivity, experimental evolution, killer virus, *S. cerevisiae*

## Abstract

Experiments on yeast cells that are hosts to a killer virus confirm that natural selection can sometimes reduce fitness.

**Related research article** Buskirk SW, Rokes AB, Lang GI. 2020. Adaptive evolution of nontransitive fitness in yeast. *eLife* **9**:e62238. doi: 10.7554/eLife.62238

It is hard to ignore the sense that life has purpose. This idea – known as teleology – is central to religious thinking. However, it is also found in many areas of human culture and scholarship that one might expect to be free from divine influence. These other areas include, somewhat surprisingly, the study of evolution. Look at the *March of Progress*, for example: in this infamous illustration a knuckle-dragging beast gradually evolves to become an erect intelligent human. Experts agree that this widely parodied image gives the wrong impression, but the feeling that evolution is progressive persists.

Perhaps the problem is the word itself. To evolve originally meant to unroll, implying the roll-out of a predetermined form (Bowler, 1975). Scientists used it to describe the embryonic development of an individual, back when it was thought that every human grew from a homunculus, a complete miniature person contained within sperm, just waiting to 'evolve' (Horder, 2010). By the mid-19th century 'evolution' had evolved to mean not just the developmental changes that occurred in individuals during their lifetimes, but directional changes observed in species across the geological timescales preserved within the fossil record. Early evolutionists, such as Lamarck, proposed teleologies in which living things are innately driven to progressively evolve more advanced adaptations. But Darwinian natural selection works without these vital forces or supernatural design, and it is notable that Darwin himself rarely used the word evolution in reference to his revolutionary theory.

Evolution, in the modern Darwinian sense, is essentially a random process. Mutations are random, but they are also heritable, so those that happen to improve their own transmission (that is, to increase fitness) will spread, resulting in adaptation. This is natural selection. But there is no direction to the process. Consider eyes, organs so complex that they fool some into thinking they must have been designed by a creator. Yet, having finally evolved this magnificent complexity, eyes will quite readily un-evolve again when their owners move into lightless caves, where vision is a useless and expensive liability.

But does natural selection not imply a particular form of progress, in that fitness itself must always increase? Not necessarily. Now, in eLife, Sean Buskirk, Alecia Rokes and Greg Lang report the results of experiments confirming that natural selection can sometimes result in a reduction of fitness (Buskirk et al., 2020).

The researchers, who are based at Lehigh University, allowed populations of yeast cells to evolve for 1000 generations, freezing live samples at regular intervals to create a ‘fossil record’ from which ancestors and descendants could be defrosted and compared. They found that the most evolved generations (those from the end of the experiment) would leave more offspring than intermediate generations (from the middle of the experiments) when both were mixed and allowed to compete directly: that is, their Darwinian fitness had increased. But when mixed with their original ancestors (from the start of the experiments), they were less fit; the original ancestors left more offspring. Yet, the intermediate generations were fitter than the original ancestors. So, while fitness did in fact increase at each step, it did not add up – together, somehow, two increases made a decrease.

To understand why, we need to know that the ancestor yeast cells were host to a common ‘killer’ virus (Figure 1). The virus encodes both a deadly toxin and resistance to that toxin, so yeast cells containing the virus are immune, but the yeast cells without are not. The virus cannot infect new host cells and is only transmitted through the offspring of its hosts. However, there is no benefit to making a toxin if all your competitors are resistant. So, as the virus populations evolved, the ability to make a worthless toxin was lost. And without the toxin, there was no advantage to having resistance to it so, eventually, resistance was also lost in the most evolved generations of yeast cells.

**Figure 1.**
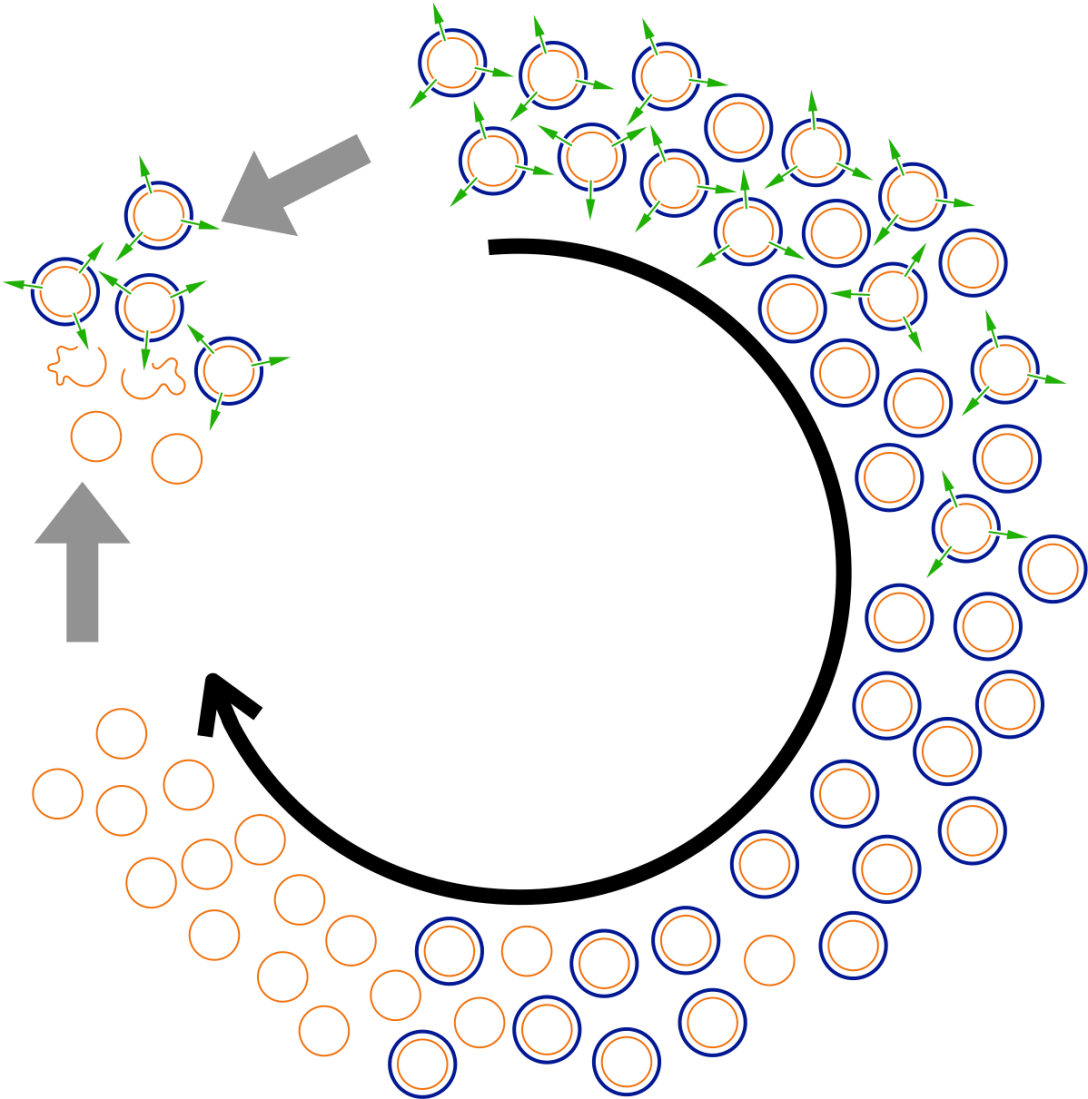
An example of evolution going round in circles. Buskirk et al. used yeast cells (thin orange circles) that are infected with a virus producing both a killer toxin (green arrows) and resistance to the toxin (thick blue circles) at the start of the experiment (represented by the 12 o’clock position). As evolution proceeds (black arrow), cells that no longer produce the toxin but are still resistant to it, take over. Eventually, these cells are replaced by cells that have lost their resistance (since resistance now provides no benefit). But when cells from the latest generation are pitted against cells from the original generation (grey arrows), the latter emerge victorious as the toxins they produce kill the former (open orange squiggles). However, cells from the latest generation can outcompete cells from intermediate generations, and cells from intermediate generations can outcompete cells from the original generations.

Thus, when cells from these generations were introduced to cells from the original generations, they succumbed to the viral toxin. Buskirk et al. were able to show that natural selection acting on the host genomes, the viral genomes, or both, drove the entire process, eventually reducing the long-term competitive fitness of the yeast. So, evolutionary changes, including fitness, are not necessarily progressive.

Is this due to having two genomes – viral and nuclear – with intertwined fates? Probably not. Take the game rock-paper-scissors as an illustration. An imaginary population of reproductive rocks might evolve into mutant pieces of paper, which would have higher fitness. But once paper has taken over, it would be replaced by descendants that evolved into scissors. Are scissors fitter than their distant ancestors, the rocks? No.

Such circular interactions – where everyone can beat someone, but everyone can also be beaten by someone else – are common in nature, both between and within species (Soliveres et al., 2018; Sinervo and Lively, 1996). But Buskirk et al. show for the first time that the different players can also replace each other within a single evolutionary lineage. We sometimes feel we are making great progress – in art, architecture, fashion, or even in the unfolding of historical events – only to recognize something from the past coming round again. Evolution seems much the same.
